# Design, Synthesis and Evaluation of Novel Phthalimide Derivatives as *in Vitro* Anti-Microbial, Anti-Oxidant and Anti-Inflammatory Agents

**DOI:** 10.3390/molecules200916620

**Published:** 2015-09-14

**Authors:** Phoebe F. Lamie, John N. Phillopes, Ahmed O. El-Gendy, Lucie Rarova, Jiri Gruz

**Affiliations:** 1 Department of Pharmaceutical Organic Chemistry, Faculty of Pharmacy, Beni Suef University, Beni Suef 62514, Egypt; E-Mail: john_philoppes@yahoo.com; 2 Department of Microbiology & Immunology, Faculty of Pharmacy, Beni Suef University, Beni Suef 62514, Egypt; E-Mail: dr.ahmed_micro@yahoo.com; 3 Laboratory of Growth Regulators & Department of Chemical Biology and Genetics, Centre of the Region Haná for Biotechnological and Agricultural Research, Institute of Experimental Botany ASCR & Palacký University, Šlechtitelů 27, 783 71 Olomouc, The Czech Republic; E-Mails: lucie.rarova@upol.cz (L.R.); jiri.gruz@upol.cz (J.G.)

**Keywords:** synthesis, phthalimides, anti-microbial, anti-oxidant, anti-inflammatory

## Abstract

Sixteen new phthalimide derivatives were synthesized and evaluated for their *in vitro* anti-microbial, anti-oxidant and anti-inflammatory activities. The cytotoxicity for all synthesized compounds was also determined in cancer cell lines and in normal human cells. None of the target derivatives had any cytotoxic activity. (*ZE*)*-*2-[4-(1-Hydrazono-ethyl)phenyl]isoindoline-1,3-dione (**12**) showed remarkable anti-microbial activity. Its activity against *Bacillus subtilis* was 133%, 106% and 88.8% when compared with the standard antibiotics ampicillin, cefotaxime and gentamicin, respectively. Compound **12** also showed its highest activities in Gram negative bacteria against *Pseudomonas aeruginosa* where the percentage activities were 75% and 57.6% when compared sequentially with the standard antibiotics cefotaxime and gentamicin. It was also found that the compounds 2-[4-(4-ethyl-3-methyl-5-thioxo-1,2,4-triazolidin-3-yl)phenyl]isoindoline-1,3-dione (**13b**) and 2-[4-(3-methyl-5-thioxo-4-phenyl-1,2,4-triazolidin-3-yl)phenyl]isoindoline-1,3-dione (**13c**) had anti-oxidant activity. 4-(*N'*-{1-[4-(1,3-Dioxo-1,3-dihydro-isoindol-2-yl)-phenyl]-ethylidene}-hydrazino)-benzenesulfonamide (**17c**) showed the highest *in vitro* anti-inflammatory activity of the tested compounds (a decrease of 32%). To determine the mechanism of the anti-inflammatory activity of **17c**, a docking study was carried out on the COX-2 enzyme. The results confirmed that **17c** had a higher binding energy score (−17.89 kcal/mol) than that of the ligand celecoxib (−17.27 kcal/mol).

## 1. Introduction

The most important biological activity properties that have been reported for phthalimide (isoindoline-1,3-dione) derivatives **1** are anti-cancer [1], anti-microbial [2,3], anti-oxidant [4] and anti-inflammatory [5]. According to the World Health Organization (WHO), infectious and parasitic diseases are still the second cause of death worldwide. This is assumed to be due to resistance to the anti-microbial agents used. There are a number of studies showing that compounds bearing a phthalimide core may be a scaffold for designing new anti-microbial agents [2]. On the other hand, oxidation results in free radicals which damage the cell via causing oxidative stress leading to inflammation [6].

A literature search of suitable nuclei of use as anti-oxidant and anti-inflammatory agents, revealed phthalimide was one of these heterocyclic compounds [1,4,5]. The chemical core of phthalimides (-CO-N(R)-CO-) shows they are hydrophobic and this increases their potential to cross biological membranes *in vivo* [7]. To increase the biological activity of phthalimide derivatives, a molecular hybridization approach was used to introduce different pharmacophore subunits such as pyrazoles, diazoles, (oxo and thioxo) triazoles, benzo- (oxazoles, imidazoles and thiazoles) and compounds with the azomethine group (Schiff basses). Earlier described synthons have been reported to have anti-microbial [8], anti-oxidant [9] and anti-inflammatory [10,11,12,13] activities, apart from other pharmacological actions like anti-convulsant [14], CNS depressant [15], anti-tumor [1,16], anti-proliferative [17] and anti-pyretic [18] effects.

**Figure 1:**
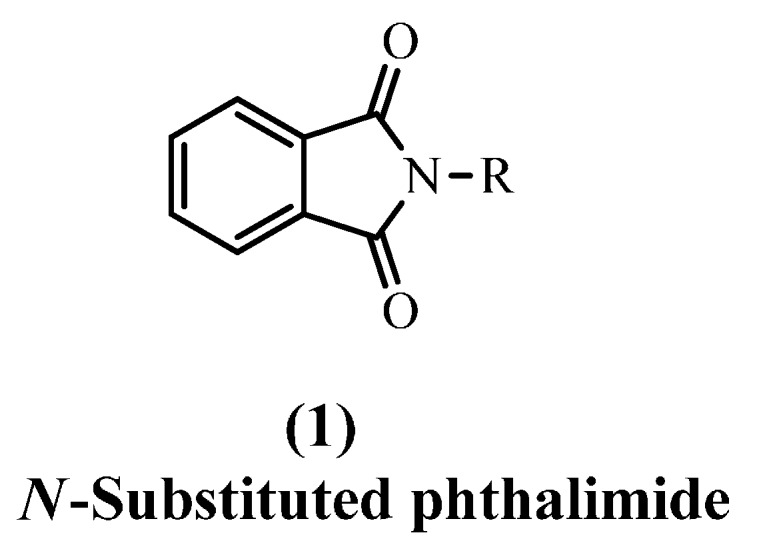

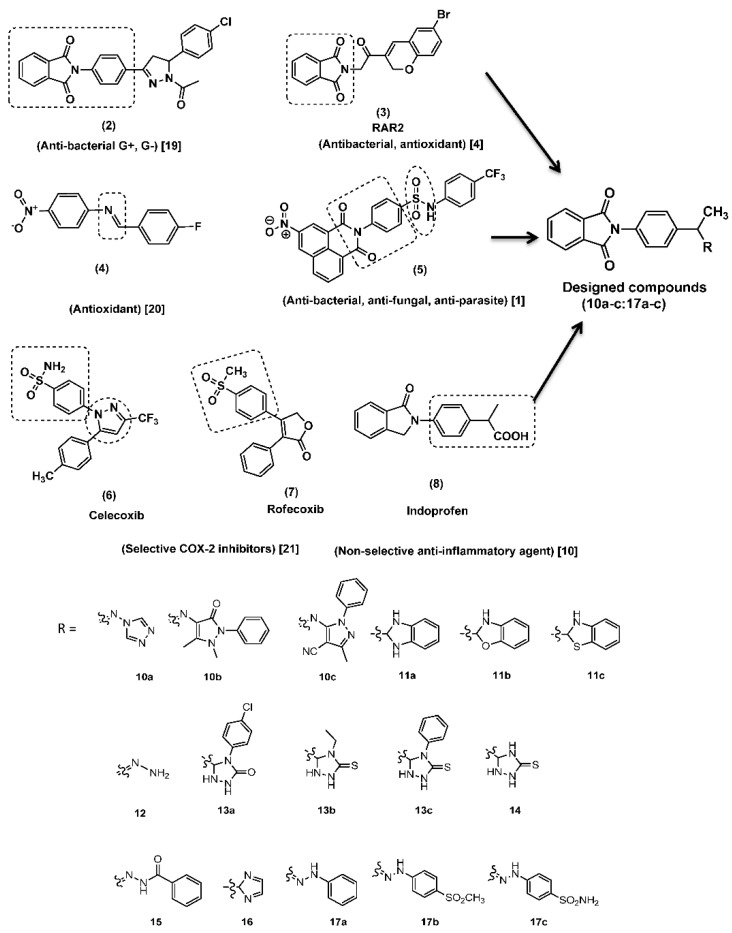
Representative examples of anti-microbial, anti-oxidant and anti-inflammatory agents and structural rationalization of the newly designed compounds.

Inspection of the compounds **2**–**8** depicted in Figure 1 led us to design and synthesize some new compounds containing mainly a phthalimide core and enhanced by certain pharmacophores. The aim was to find new agents with anti-microbial, anti-oxidant and anti-inflammatory effects. All the obtained new derivatives were tested as anti-microbial agents (G+, G− bacteria and fungi) and studied for their anti-oxidant and anti-inflammatory activities using *in vitro* methods, (Figure 1).

## 2. Results and Discussion

### 2.1. Chemical Synthesis

**Scheme 1:**
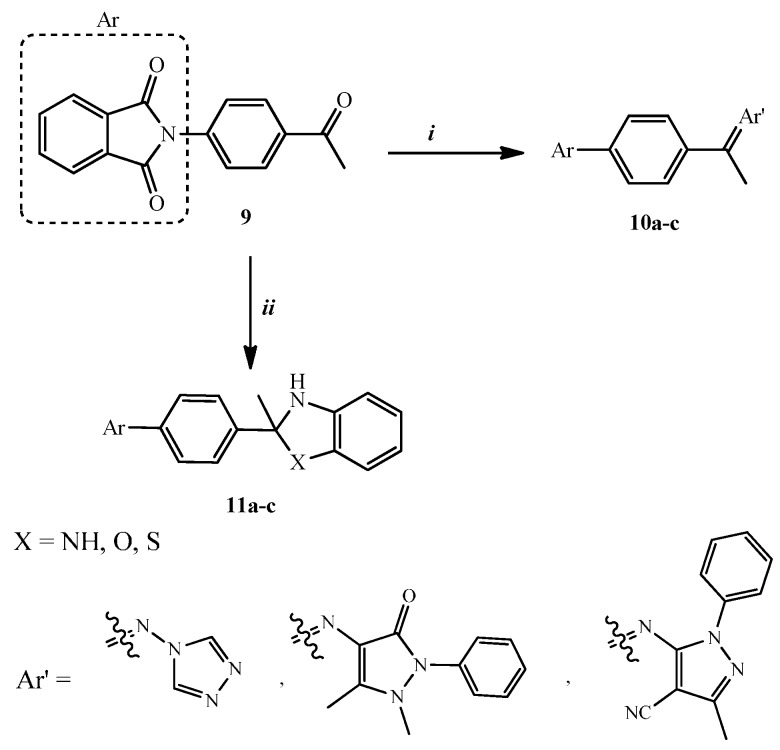
Synthesis of compounds **10a**–**c** and compound **11a**–**c**. *Reagents and conditions:* (***i***) ArʹNH_2_, absolute ethanol, reflux, 8 h, (32%–44%); (***ii***) *o*-phenylenediamine, *o*-aminophenol or *o*-aminothiophenol, absolute ethanol/glacial acetic acid, reflux, 12 h, (26%–46%).

**Scheme 2:**
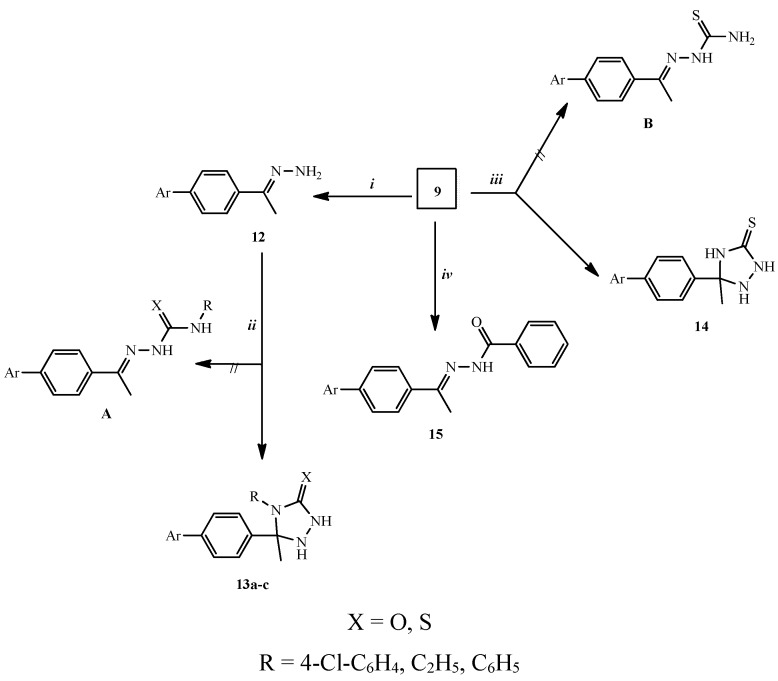
Synthesis of triazole derivatives **13a**–**c**, **14** and compound **15**. *Reagents and conditions:* (***i***) NH_2_NH_2_, dioxane, reflux, 2 h, (24%); (***ii***) RNCX, dioxane, reflux, 5 h, (39%–47%); (***iii***) NH_2_NHCSNH_2_, dioxane, reflux, 3 h, (33%); (***iv***) PhCONHNH_2_, dioxane, reflux, 7 h, (48%).

**Scheme 3:**
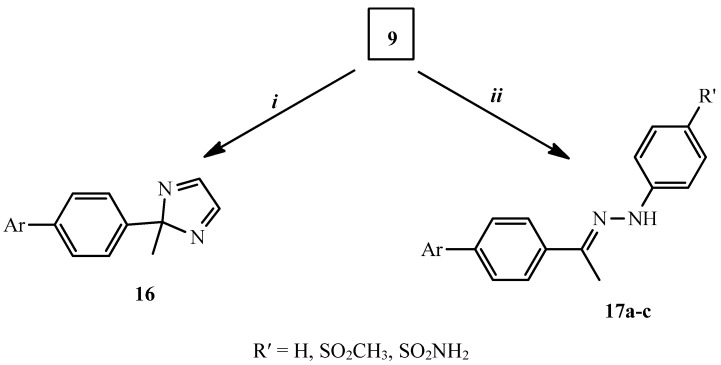
Synthesis of compounds **16** and **17a**–**c**. *Reagents and conditions*: (***i***) NH_2_CH_2_CH_2_NH_2_, dioxane, reflux, 4 h, (26%); (***ii***) 4-RʹPhNHNH_2_.HCl, absolute ethanol, reflux, 5–8 h, (49%–52%).

The synthetic procedures adopted to obtain the target compounds are depicted in Scheme 1, Scheme 2 and Scheme 3. Reaction of 2-(4-acetyl-phenyl)-isoindoline-1,3-dione (**9**), which was prepared according to the reported method [19], with equimolar amounts of primary amine derivatives, namely 1-aminotriazole, 4-amino-1,5-dimethyl-2-phenyl-1,2-dihydro-pyrazol-3-one (4-aminophenazone) and 5-amino-3-methyl-1-phenyl-1*H*-pyrazole-4-carbonitrile in absolute ethanol at reflux temperature for 8 h to give isoindoline-1,3-dione derivatives **10a**–**c**. The structure of compounds **10a**–**c** was investigated by elemental and spectral analyses. The IR spectrum of **10c** revealed a C≡N stretching band at ῡ 2212 cm^−^^1^. The ^1^H-NMR spectrum of **10b** exhibited three singlet peaks due to two CH_3_ and N-CH_3_ protons at δ 2.1, 2.5 and 2.63, respectively. Moreover, ^13^C-NMR spectrum of **10c** showed a peak at δ 115.04 corresponding to C≡N.

Compounds **11a**–**c** were prepared by the reaction of 2-(4-acetyl-phenyl)-isoindoline-1,3-dione (**9**) with *o*-amino derivatives namely, *o*-phenylenediamine, *o*-aminophenol and *o*-aminothiophenol in refluxing absolute ethanol containing few drops of glacial acetic acid yielding **11a**–**c** in good yields. Spectroscopic data (IR, ^1^H-NMR, ^13^C-NMR and MS) and elemental analysis of compounds **11a**–**c** confirmed their structures. The ^1^H-NMR spectra of **11a**–**c** exhibited D_2_O exchangeable singlet peaks at δ 3.35–4.45 due to the NH proton, confirming that **11a**–**c** exists as cyclic benzimidazole, dihydrobenzoxazole and dihydrobenzothiazole with isoindoline-1,3-dione. Another NH proton appeared in **11a** at δ 7.65 corresponding to a benzimidazole that was exchanged with D_2_O (Scheme 1).

Treatment of **9** with excess hydrazine hydrate 99% in dioxane at reflux temperature afforded 2-[4-(1-hydrazonoethyl)phenyl]isoindoline-1,3-dione (**12**, 45% yield). All collected data for compound **12** were in accord with the assumed structure. Thus, the absence of C=O group of the parent **9** and the observation of forked peak at ῡ 3317 and 3235 cm^−1^ corresponding NH_2_ group in IR spectrum of **12** confirmed its structure. Further the ^1^H-NMR spectrum of **12** showed D_2_O exchangeable protons at δ 5.79 corresponding to NH_2_ protons. Moreover, the absence of the C=O observed in the starting material **9** in ^13^C-NMR spectrum of **12** confirmed the predicted structure of **12**.

Compound **12** was treated with isocyanate and/or isothiocyanate derivatives, e.g., *p*-chlorophenylisocyanate, ethylisothiocyanate and phenylisothiocyanate in dioxane at reflux temperature to provide the corresponding oxo(or thioxo)triazole derivatives **13a**–**c** rather than semicarbazide or thiosemicarbazide analogues **A**. The structures of compounds **13a**–**c** were investigated by elemental and spectral analyses. The ^13^C-NMR spectra of **13a**–**c** revealed peaks at δ 70.80–90.03 due to the CH_3_-*C* of the triazole ring which is not present in the open form **A**.

The triazole ring was also assembled via reaction of compound **9** with a thiosemicarbazide derivative yielding **14** rather than the open form **B**. The structure of compound **14** was established through, spectroscopic and elemental analyses data. The ^13^C-NMR spectrum of **14** showed the presence of the CH_3_-*C* moiety of the thioxotriazole ring at δ 66.80 that was absent in the starting material **9** or in the thiosemicarbazide derivative **B**, thus confirming the putative structure **14**.

Treatment of **9** with a molar equivalent of benzohydrazide derivative in dioxane at reflux temperature for 7 h, gave *N*ʹ-{1-[4-(1,3-dioxoindolin-2-yl)phenyl]ethylidene}benzohydrazide (**15**) as a yellow solid in 48% yield. The structure of compound **15** was confirmed by elemental and spectroscopic analyses. The IR spectrum of **15** showed NH stretching at ῡ 3330 cm^−1^ apart from a C=O band at ῡ 1672 cm^−1^. Moreover, the ^1^H-NMR spectrum of **15** revealed the presence of two singlet peaks at δ 2.56 and 2.64 due to the *syn* and *anti* CH_3_ groups around the CH_3_-C=N double bond. Also, in the mass spectrum of **15** the molecular ion [M^+^] (*m*/*z* 383) corresponding to the formula C_23_H_17_N_3_O_3_ was observed (Scheme 2).

Introducing a diazole moiety to compound **16** was achieved by heating **9** with an equimolar quantity of ethylenediamine in refluxing dioxane for 4 h. The IR spectrum of **16** revealed the absence of the C=O group of the parent compound **9**. Moreover, aromatization of the diazole ring was confirmed from the ^1^H-NMR spectrum that showed two CH diazole ring protons as doublets at δ 6.56 and 7.66 with a coupling constant of 8.4 Hz. The mass spectrum of **16** exhibited a molecular ion peak [M^+^] at (*m*/*z* 303) confirming its molecular formula C_18_H_13_N_3_O_2_.

Treatment of **9** with hydrochloride salt of phenylhydrazine, *p*-methanesulfonylphenylhydrazine or *p*-aminosulfonylphenylhydrazine in a molar ratio (1:1) in refluxing absolute ethanol gave **17a**–**c** in excellent yields. All data for compounds **17a**–**c** were consistent with the proposed structures. Thus, the absence of the C=O group of the parent **9** in IR spectra of **17a**–**c** and the appearance of NH (in **17a**–**c**) and NH_2_ (in **17c**) stretching bands at ῡ 3428–3258 cm^−1^ confirmed the structures of **17a**–**c**. Moreover, the *syn* and *anti* CH_3_ protons were observed at δ 2.30–2.36 and 2.51–2.63. Further, the NH proton singlet was recorded at δ 9.87–9.98 that was exchangeable with D_2_O confirmed structure of the **17a**–**c** (Scheme 3).

### 2.2. Biological Evaluation

#### 2.2.1. Anti-Microbial Activity

**Figure 2:**
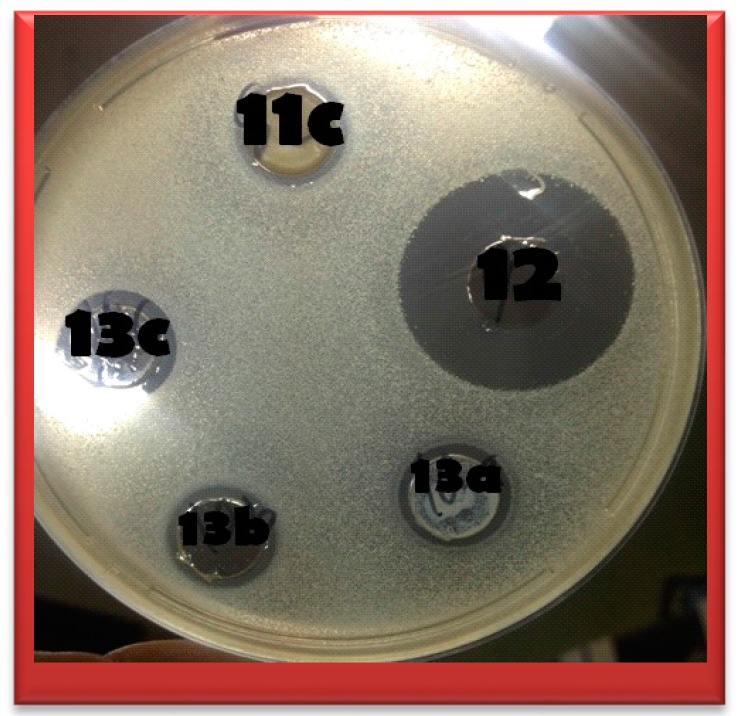
Examples of some activities for compounds **11c**, **12**, **13a**, **13b** and **13c** against *Bacillus subtilis*.

Different species of microorganisms have varying degrees of susceptibility to antimicrobials. Further, the pathogenic microbes may develop drug resistance to a particular type of antimicrobial agent on prolonged use. Hence, the antimicrobial sensitivity tests are very useful to determine the level of antimicrobial activity of a particular chemical compound on certain pathogenic microorganisms using agar well diffusion method, (Figure 2).

**Table 1:** The zone of inhibition (mm) for test compounds *^#^.

	Gram Positive Rods			Gram Positive Cocci			Gram Negative Rods				Yeast
	*Bacillus subtilis*	*Listeria inocua* LMG 2710	*Mycobacterium pheli*	*Enterococcus faecalis*	*Sarcina lutea*	*Staphylococcus aureus* LMG 3242	*Escherichia coli* ATCC 25922	*Escherichia coli* ATCC 5087	*Psedomonas aeruginosa*	*Proteus vulgaris*	*Candida albicans*
**10a**	13	-	-	-	-	14	-	-	-	-	14
**10b**	13	15	14.5	13	-	15	-	-	-	-	16
**10c**	-	15	15	-	-	-	-	-	12	-	14
**11a**	15	13	13	16	-	14.5	13	-	13	-	14
**11b**	15	13	13	16.5	-	14	12	-	12	-	13
**11c**	-	17	-	-	-	14.5	-	-	13	-	20
**12**	26	17	23	17.5	25	16	17	17	25	-	21
**13a**	13	14	-	15.5	-	13	-	-	-	-	-
**13b**	13	15.5	13	14	14	13	-	-	-	-	14
**13c**	14	16	16	13.5	17.5	13	-	-	13	-	13
**14**	16	15	15.5	15	18	-	-	-	13	12	14
**15**	13	-	-	13.5	-	-	-	-	11	-	17.5
**16**	12	12	-	13	-	-	-	-	12	-	18
**17a**	11	17	11	12	-	-	-	-	11	12	16.5
**17b**	13	11	-	13	-	-	-	-	12	-	12
**17c**	-	16	-	13	-	-	-	-	13	-	15
**AMP ^(a)^**	22	30	42	30	54	31	38	44	-	-	-
**CTX ^(b)^**	25	21	40	22	48	25	42	52	30	31	-
**CN ^(c)^**	28	31	36	20	40	24	34	40	36	39	-
**FLU ^(d)^**	-	-	-	-	-	-	-	-	-	-	40
**TIO ^(e)^**	-	-	-	-	-	-	-	-	-	-	36

* All synthesized compounds and control anti-microbial were dissolved in DMSO to give a final concentration of 10 mg/mL and then a 60 µL of each was inoculated into cup in agar media. ^#^ Incubation temperature was 37 ± 1 °C for 24 h. ^(a)^ AMP: Ampicillin; ^(b)^ CTX: Cefotaxime; ^(c)^ CN: Gentamicin; ^(d)^ FLU: Fluconazole; ^(e)^ TIO: Tioconazole.

**Table 2:** MIC of samples against different microorganisms (µg/mL) *^#^.

Compound No.	*Proteus vulgaris*	*Salmonella typhi*	*Pseudomonas aeruginosa* 9027	*E. coli* 25927	*E. coli* 5087	*Sarcina lutea*	*Enterococcus faecalis* OS4	*Mycobacterium pheli*	*Lactobacillus sakei* LMG 2313	*Bacillus subtilis*	*Listeria innocua* LMG 2710	*Staphylococcus aureus* LMG 3242	*Staphylococcus aureus* ATCC 43300	*Candida albicans* ATCC 60931
**10b**	na	na	na	na	na	na	na	na	na	na	na	na	na	na
**11a**	na	na	na	na	na	na	100	50	50	na	25	50	na	na
**11b**	na	na	na	na	na	na	na	na	50	na	na	na	na	na
**11c**	na	na	na	na	na	na	na	na	na	na	100	na	na	100
**12**	na	na	100	na	na	50	50	25	200	25	100	na	na	100
**13c**	200	na	200	200	200	na	200	na	200	200	200	200	200	na
**14**	na	na	na	na	na	na	na	na	na	na	na	na	na	na
**16**	na	na	na	na	na	na	na	na	na	na	na	na	na	na
**17a**	na	na	na	na	na	na	na	na	na	na	na	na	na	25

* All tested compounds and controls were used in a final concentration ranging from (200 µg/mL–0.7 µg/mL). ^#^ Incubation temperature was 37 ± 1 °C for 24 h. na: (not active >200 unit).

*Candida albicans*, *Listeria innocua*, *Bacillus subtilis* and *Enterococcus faecalis* were the most susceptible microorganisms toward the majority of our synthetic chemical compounds, while *Escherichia coli*, *Proteus vulgaris* and *Sarcine lutea* were the most resistant bacteria. Based on the antimicrobial sensitivity determinations, all of our compounds showed marked activity against at least two out of the six tested Gram positive bacteria, where the order of activity was as follows; compound **12** showed the highest activity followed by **13c**, **13b**, **14**, (**11a** = **11b**), **10b**, **13a**, **17a**, (**16** = **17b**), **11c**, **10c**, **17c**, **15** and finally **10a**. Compounds **12**, **13c** and **13b** were able to inhibit all six tested Gram positive bacteria. Only compound **12** showed marked activity against *Bacillus subtilis* where the percentage activities were 133%, 106% and 88.8% when compared with the standard antibiotics ampicillin, cefotaxime and gentamicin, respectively. Activity against Gram negative bacteria was weak, and only compound **12** was able to inhibit 3/4 tested organisms. Compound **12** also showed its highest activities in Gram negative bacteria against *Pseudomonas aeruginosa* where the percentage activities were 75% and 57.6% when compared with the standard antibiotics cefotaxime and gentamicin, respectively. The order of activity against Gram negative bacteria was as follows; **12**, **11a**, **14**, **11b**, **17a**, (**11c** = **13c** = **17c**), (**10c** = **16** = **17b**) and finally **15** while compounds **10b**, **13a**, **13b** and **10a** failed to show any activity against any of the tested Gram negative bacteria at the tested concentrations. All compounds apart from **13a** showed activity against *Candida albicans* in the following order of activity: **12**, **11c**, **16**, **15**, **17a**, **10b**, **17c**, (**10a** = **10** = **11a** = **13b** = **14**), (**11b** = **13c**) and **17b** (Table 1 and Table 2).

#### 2.2.2. Anti-Oxidant Activity

Oxygen radical absorbance capacity (ORAC) is the ability of compounds to scavenge free peroxy radicals *in vitro*. While **10a**, **10c** and **16** activities were below detection limits (<0.1), **13b** and **13c** were 18 × (18.385 ± 0.857) and 14 × times (13.506 ± 0.819) more active than Trolox, the hydrophilic derivative of vitamin E (Table 3).

**Table 3:** Oxygen radical absorbance capacity given as ratio between compound and Trolox on an equimolar basis. Data are expressed as mean ± SD (*n* = 4).

Compound	ORAC (Compound/Trolox)
**10a**	<0.1
**10b**	0.112 ± 0.007
**10c**	<0.1
**11a**	0.751 ± 0.081
**11b**	0.116 ± 0.004
**11c**	0.183 ± 0.01
**12**	8.116 ± 0.343
**13a**	1.169 ± 0.118
**13b**	18.385 ± 0.857
**13c**	13.506 ± 0.819
**14**	1.511 ± 0.045
**15**	0.192 ± 0.005
**16**	<0.1
**17a**	1.839 ± 0.035
**17b**	0.129 ± 0.005
**17c**	0.374 ± 0.007

#### 2.2.3. Anti-Inflammatory Activity

**Figure 3:**
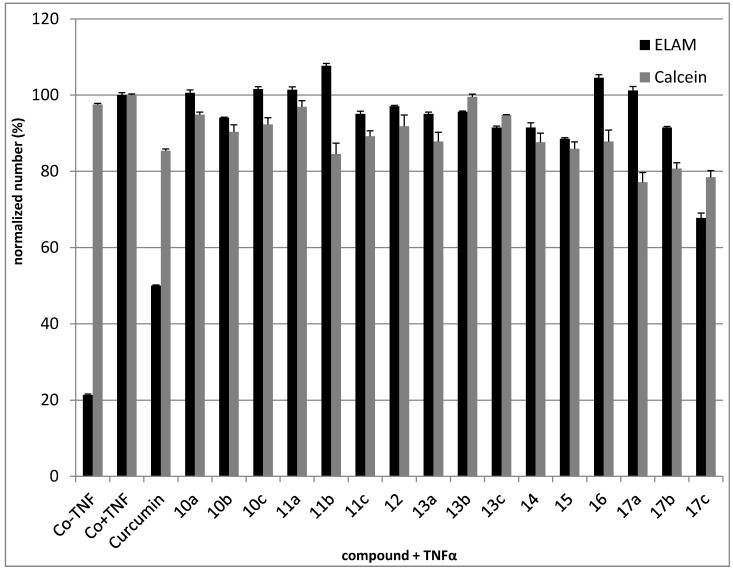
E-Selectin (ELAM) expression in TNFα -induced HUVECs compared to cytotoxicity determined by Calcein AM. Curcumin (10 μM) was used as a positive control.

The *in vitro* anti-inflammatory properties of the phthalimide derivatives were studied using ELISA in pretreated Human Umbilical Vein Endothelial Cells (HUVEC) where these compounds could inhibit NF-κB. E-selectin (ELAM) expression was induced by TNFα, which is indicative of NF-κB activation. The observed reduction of ELAM expression on treatment of HUVECs with 30 µM or 50 µM of phthalimide derivatives was significant only for **17c**—*p*-aminosulfone derivative—(decrease of 32%). Slight inhibition of ELAM expression and also cell viability was measured for **10b**, **11c**, **12**, **13a**, **13b**, **13c**, **14**, **15**, **17b**. The results showed confirmed that the NF-κB pathway was targeted by the phthalimide derivatives (Figure 3).

1 × 10^4^ HUVECs/well were seeded into 96-well plates and grown to confluence. 50 µM of phthalimide derivatives were added 30 min prior to application of 10 ng/mL TNFα for another 4 h. The cells were then fixed and ELAM levels analysed by ELISA. At the same time, the compounds were analysed by Calcein AM assay to monitor non-specific substance toxicity.

#### 2.2.4. Cytotoxic Activity

Sixteen phthalimide derivatives (**10a**–**c**, **11a**–**c**, **12**, **13a**–**c**, **14**, **15**, **16**, **17a**–**c**) were tested for cytotoxicity in three different cancer cell lines derived from various tumours (CEM, MCF7, HeLa), normal human fibroblasts (BJ) and human umbilical vein endothelial cells (HUVEC). Treatment of cancer cell lines and fibroblasts with tested compounds for 72 h resulted in no loss of viability (data not shown). After 24 h, the phthalimide derivatives showed no cytotoxicity towards HUVECs (data not shown).

### 2.3. Molecular Modeling

Compound **17c** had the highest anti-inflammatory effects of all tested compounds. Given that the most important enzymatic system in inflammation is the cyclooxygenase system [20], a molecular modelling study was carried out to investigate the binding conformation for **17c** and the active binding site of the COX-2 enzyme. The molecular docking study was done using the crystal structure of COX-2 (protein database [PDB] entry: ID 3LN1] [21], with compound **17c** (containing COX-2 pharmacophore moiety –SO_2_NH_2_ and celecoxib (**6**) as the reference ligand. The modelling software which was used for this study was the MOE version 2008.10 (Molecular Operating Environment-Montreal, QC, Canada) [22].

**Figure 4:**
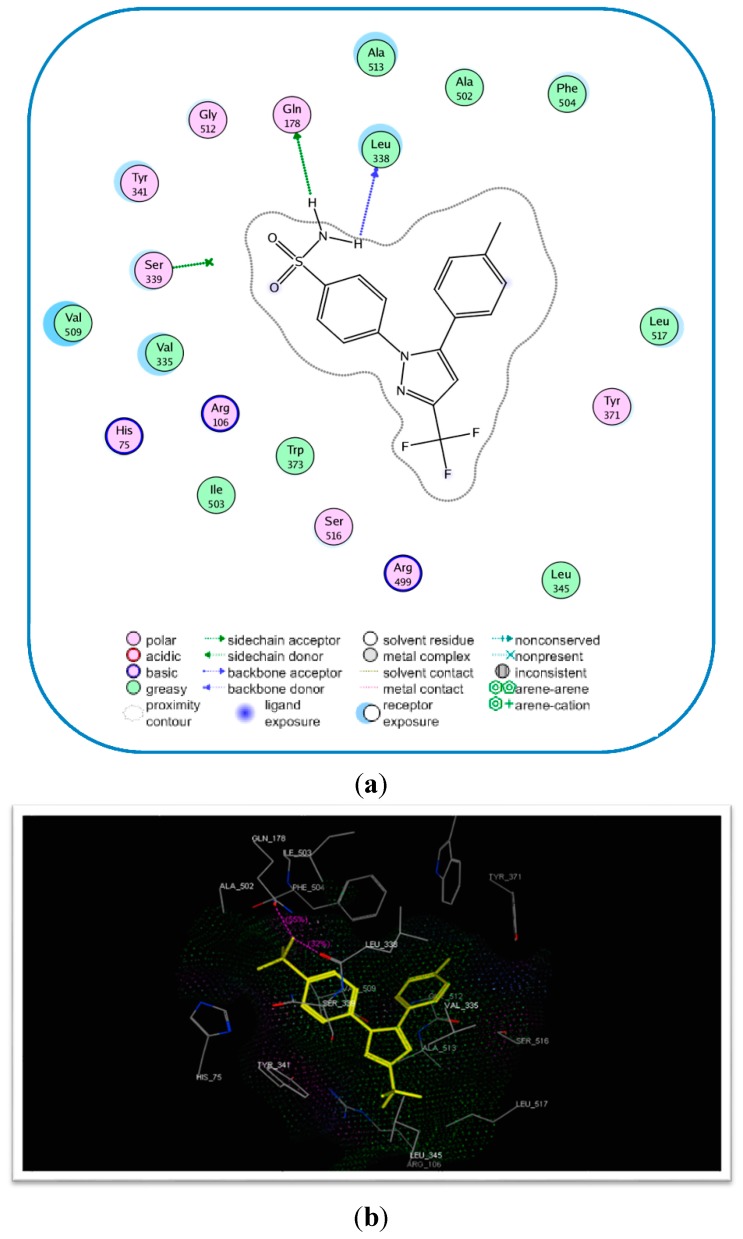

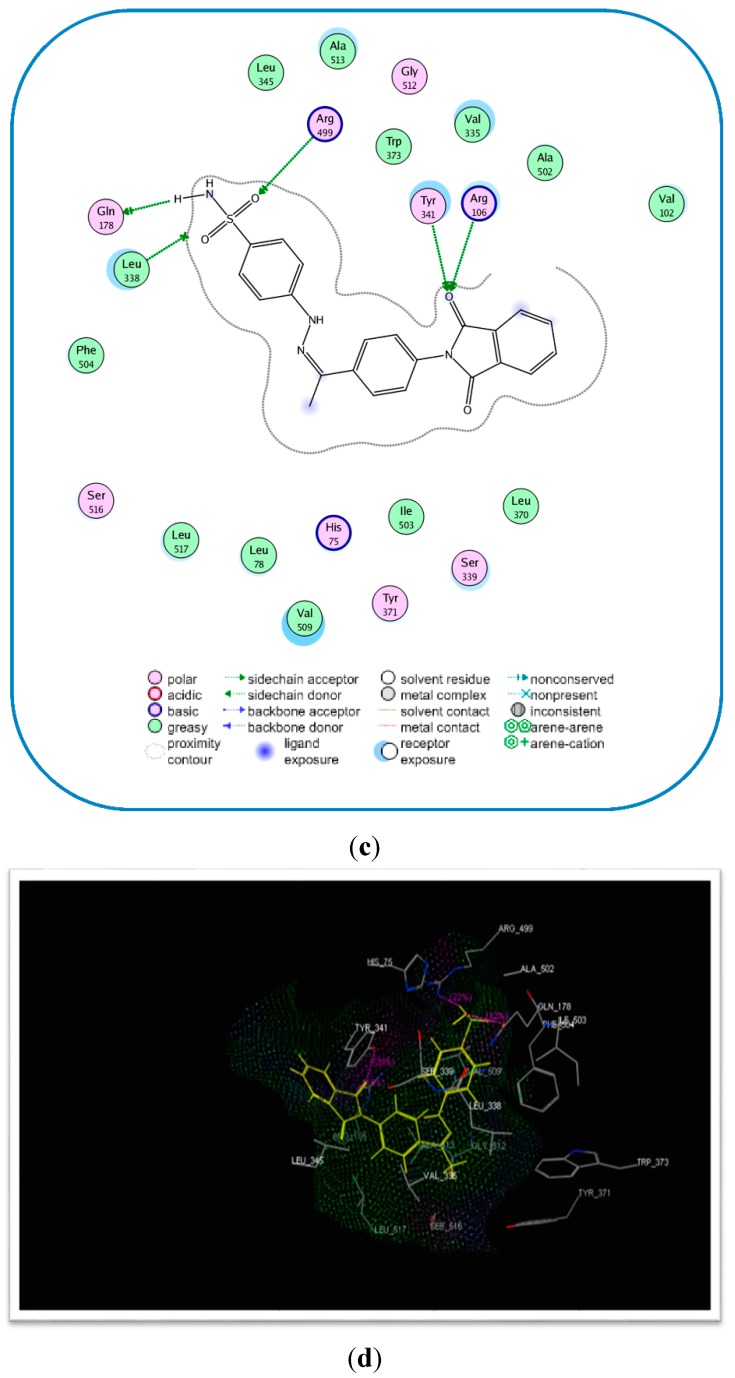
Binding of celecoxib (**6**) and the most active compound **17c** inside COX-2 active site. (**a**) 2D Interaction for binding of celecoxib (**6**) inside COX-2 active site, the most important amino acids are shown together with their respective numbers; (**b**) 3D interaction of celecoxib (**6**); (**c**) 2D interaction for binding of the most active compound **17c** inside COX-2 active site, the most important amino acids are shown together with their respective numbers; (**d**) 3D interaction of **17c**.

Celecoxib as the ligand used in this study, was flexibly docked to the binding site of the COX-2 enzyme, and the docking conformation that was represented by the lowest energy score value was selected. The newly synthesized compound **17c** was docked. The docking study showed that **17c** occupied the COX-2 binding site compared to celecoxib (**6**), (Figure 4).

The binding energy for compound **17c** was −17.89 kcal/mol compared to −17.27 kcal/mol for celecoxib (**6**). Moreover, compound **17c** exhibited four hydrogen bonding interactions with Tyr341 (C=O), Arg106 (C=O), Arg499 (S=O), and Gln178 (NH) and amino acids with hydrogen bond lengths (2.75, 2.77, 2.96, and 2.21 Å, respectively), (Table 4 and Figure 4).

**Table 4:** Molecular modelling data for compound **17c** and celecoxib (**6**) during docking in the COX-2 (PDB: ID3LN1) active site.

Compound No.	E-Refine (kcal/mol)	No. of Hydrogen Bonds	Hydrogen Bonding Residues	Distance (Å)
**17c**	−17.89	4	Tyr341 (C=O) Arg106 (C=O) Arg499 (S=O)	2.75 2.77 2.96
**Celecoxib (6)**	−17.27	2	Gln178 (NH) Leu338 (NH) Gln178 (NH)	2.21 2.63 2.55

## 3. Experimental Section

### 3.1. General Information

Melting points were determined on an Electrothermal digital melting point apparatus and are uncorrected. (IR spectra were recorded on an R 435 spectrophotometer (Middlton, Madison West, WI, USA) and values were reported in cm^−1^. ^1^H-NMR and ^13^C-NMR were carried out on Bruker Advance III 400 MHz spectrophotometer (Bruker BioSpin AG, Fällanden, Switzerland) for ^1^H and 100 MHz for ^13^C with BBFO Smart Probe and Bruker 400 AEON Nitrogen-Free Magnet, using TMS as an internal standard and chemical shifts were recorded in ppm on δ scale, Faculty of Pharmacy, Beni Suef University, Egypt. The electron impact (EI) mass spectra were recorded on a Hewlett Packard 5988 spectrometer (Palo Alto, CA, USA), Microanalyses for C, H and N were carried out on Perkin-Elmer 2400 analyzer (Perkin-Elmer, Norwalk, CT, USA) at the Micro analytical unit of Cairo University, Egypt, and all compounds were within ±0.4% of the theoretical values. Thin-layer chromatography (TLC) was performed on Merck (Darmstadt, Germany ) TLC aluminium sheets silica gel 60 F_254_ with detection by UV quenching at 254 nm to follow the course of reactions and to check the purity of products. All reagents and solvents were purified and dried by standard techniques.

#### 3.1.1. General Procedure for the Synthesis of Compounds **10a**–**c**

A mixture of **9** (2.65 g, 0.01 mol) and the appropriate primary amine (0.01 mol) was refluxed in absolute ethanol (30 mL) for 8 h. The solid separated on hot was filtered, dried and crystallized from dioxane.

*(ZE)-2-{4-[1-(4H-1,2,4-Triazol-4-ylimino)ethyl]phenyl}isoindoline-1,3-dione* (**10a**). Yield 32%; m.p. 222–224 °C; IR (KBr, cm^−1^): 1785 and 1714 (2C=O, isoindoline-1,3-dione); ^1^H-NMR (DMSO-*d*_6_) δ 2.63 (s, 3H, CH_3_), 7.64–7.89 (m, 2H, Ar-H), 7.94 (s, 2H, Ar-H), 7.99–8.01 (m, 3H, Ar-H), 8.10–8.24 (m, 3H, Ar-H); ^13^C-NMR (DMSO-*d*_6_) δ 13.50, 124.03, 124.50, 127.54, 129.24, 131.98, 135.33, 136.28, 136.49, 158.80, 167.13; MS (*m*/*z*, %): 331 (M**^+^**, 1.77%), 64 (100%); Anal. Calcd. for C_18_H_13_N_5_O_2_: C, 65.25; H, 3.95; N, 21.14. Found: C, 65.03; H, 4.14; N, 21.30%.

*(ZE)-2-{4-[1-(1,5-Dimethyl-3-oxo-2-phenyl-2,3-dihydro-1H-pyrazol-4-ylimino)ethyl]phenyl}isoindoline-1,3-dione* (**10b**). Yield 44%; m.p. 218–220 °C; IR (KBr, cm^−1^): 1784 and 1714 (2C=O, isoindoline-1,3-dione), 1679 (C=O); ^1^H-NMR (DMSO-*d*_6_) δ 2.1 (s, 3H, CH_3_), 2.50 (s, 3H, CH_3_), 2.63 (s, 3H, CH_3_), 7.64–7.89 (m, 3H, Ar-H), 7.95–7.98 (m, 7H, Ar-H), 8.00–8.15 (m, 3H, Ar-H);^13^C-NMR (DMSO-*d*_6_) δ 15.10, 16.50, 39.32, 122.80, 123.94, 127.29, 127.51, 128.38, 128.80, 129.24, 132.01, 133.29, 135.24, 137.20, 138.08, 150.20, 158.80, 160.70, 167.36; MS (*m*/*z*, %): 450 (M**^+^**, 2.40%), 64 (100%); Anal. Calcd. for C_27_H_22_N_4_O_3_: C, 71.99; H, 4.92; N, 12.44. Found: C, 72.13; H, 4.85; N, 12.29%.

*(ZE)-5-{1-[4-(1,3-Dioxoisoindolin-2-yl)phenyl]ethylidineamino}-3-methyl-1-phenyl-1H-pyrazole-4-carbonitrile* (**10c**). Yield 36%; m.p. 232–234 °C; IR (KBr, cm^−1^): 2212 (C≡N), 1785 and 1713 (2C=O, isoindoline-1,3-dione);^1^H-NMR (DMSO-*d*_6_) δ 2.1 (s, 3H, CH_3_), 2.63 (s, 3H, CH_3_), 7.64 (d, 3H, *J* = 8 Hz, Ar-H), 7.96–8.09 (m, 7H, Ar-H), 8.11 (d, 3H, *J* = 8 Hz, Ar-H); ^13^C-NMR(DMSO-*d*_6_) δ 11.60, 18.40, 92.51, 115.04, 123.80, 123.94, 124.04, 126.20, 127.54,129.25, 131.98, 132.20, 135.34, 136.30, 136.49, 150.20, 150.40, 158.40, 167.13; MS (*m*/*z*, %): 445 (M^+^, 1.47%), 70 (100%); Anal. Calcd. for C_27_H_19_N_5_O_2_: C, 72.80; H, 4.30; N, 15.72. Found: C, 72.68; H, 4.42; N, 15.63%.

#### 3.1.2. General Procedure for the Synthesis of Compounds **11a**–**c**

A mixture of **9** (2.65 g, 0.01 mol) and the corresponding *o*-amino derivative (0.01 mol) was refluxed in absolute ethanol (30 mL) containing a catalytic amount of glacial acetic acid (0.5 mL) for 12 h. The solid separated on hot was filtered, dried and crystallized from DMF.

*2-[4-(2-Methyl-2,3-dihydro-1H-benzo[d]imidazol-2-yl)phenyl]isoindoline-1,3-dione* (**11a**). Yield 46%; m.p. 292–294 °C; IR (KBr, cm^−1^): 3447 (broad band, 2NH), 1785 and 1714 (2C=O, isoindoline-1,3-dione); ^1^H-NMR (DMSO, *d*_6_) δ 2.09 (s, 3H, CH_3_), 3.35 (s, 1H, NH, D_2_O exchangeable), 7.22–7.46 (m, 3H, Ar-H), 7.61–7.63 (m, 3H, Ar-H), 7.65 (s, 1H, NH, D_2_O exchangeable), 7.71, 7.72 (dd, 2H, *J* = 3.2 Hz, 3.2 Hz, Ar-H), 7.92, 8.01 (dd, 1H, *J* = 3.2 Hz, 3.2 Hz, Ar-H), 8.09–8.24 (m, 3H, Ar-H); ^13^C-NMR (DMSO-*d*_6_) δ 26.30, 74.70, 112.91, 117.70, 120.20, 124.03, 127.34, 128.09, 129.75, 131.02, 132.30, 136.72, 169.08; MS (*m*/*z*, %): 355 (M**^+^**, 0.17%), 77 (100%); Anal. Calcd. for C_22_H_17_N_3_O_2_: C, 74.35; H, 4.82; N, 11.82. Found: C, 74.13; H, 4.62; N, 11.80%.

*2-[4-(2-Methyl-2,3-dihydrobenzo[d]oxazol-2-yl)phenyl]isoindoline-1,3-dione* (**11b**). Yield 26%; m.p. 229–231 °C; IR (KBr, cm^−1^): 3470 (NH), 1786 and 1713 (2C=O, isoindoline-1,3-dione); ^1^H-NMR (DMSO-*d*_6_) δ 2.46 (s, 3H, CH_3_), 4.38 (s, 1H, NH, D_2_O exchangeable), 7.64 (d, 3H, *J* = 8 Hz, Ar-H), 7.99–8.00 (m, 6H, Ar-H), 8.11 (d, 3H, *J* = 8 Hz, Ar-H); ^13^C-NMR (DMSO-*d*_6_) δ 27.32, 98.21, 113.05, 114.01, 124.03, 126.90, 127.54, 128.20, 129.24, 131.99, 132.50, 135.33, 136.29, 136.50, 142.60, 167.13; MS (*m*/*z*, %): 356 (M**^+^**, 0.36%), 309 (100%); Anal. Calcd. for C_22_H_16_N_2_O_3_: C, 74.15; H, 4.53; N, 7.86. Found: C, 74.31; H, 4.62; N, 7.80%.

*2-[4-(2-Methyl-2,3-dihydrobenzo[d]thiazol-2-yl)phenyl]isoindoline-1,3-dione* (**11c**). Yield 29%; m.p. >300 °C; IR (KBr, cm^−1^): 3456 (NH), 1781 and 1714 (2C=O, isoindoline-1,3-dione); ^1^H-NMR (DMSO-*d*_6_) δ 2.50 (s, 3H, CH_3_), 4.45 (s, 1H, NH,D_2_O exchangeable), 7.19 (t, 1H, *J* = 7.2 Hz, Ar-H), 7.28 (t, 1H, *J* = 7.2 Hz, Ar-H), 7.50–7.52 (m, 2H, Ar-H), 7.56–7.65 (m, 2H, Ar-H), 7.67–7.94 (m, 4H, Ar-H), 7.98 (d, 2H, *J* = 8.4 Hz, Ar-H). ^13^C-NMR (DMSO-*d*_6_) δ 27.32, 66.81, 122.48, 123.96, 124.03, 127.53, 127.71, 127.76, 129.24, 129.36, 131.98, 135.25, 135.33, 136.28, 136.49, 167.13; MS (*m*/*z*, %): 372 (M**^+^**, 8.50%), 370 (100%); Anal. Calcd. for C_22_H_16_N_2_O_2_S: C, 70.95; H, 4.33; N, 7.52. Found: C, 70.73; H, 4.35; N, 7.31%.

#### 3.1.3. Synthesis of (*ZE*)-2-[4-(1-Hydrazonoethyl)phenyl]isoindoline-1,3-dione (**12**)

To a solution of compound **9** (2.65 g, 0.01 mol) in dioxane (30 mL), hydrazine hydrate 99.99% (0.55 g, 0.011 mol) was added and the reaction mixture was heated at reflux temperature for 2 h. The solid separated while hot was filtered, dried and crystallized from DMF. Yield 24%; m.p. >300 °C; IR (KBr, cm^−1^): 3317, 3235 (NH_2_), 1785 and 1714 (2C=O, isoindoline-1,3-dione); ^1^H-NMR (DMSO-*d*_6_) δ 2.23 (s, 3H, CH_3_), 5.79 (s, 2H, NH_2_, D_2_O exchangeable), 7.81–7.99 (m, 4H, Ar-H), 8.07–8.14 (m, 4H, Ar-H); ^13^C-NMR (DMSO-*d*_6_) δ 14.46, 113.60, 125.67, 126.24, 127.13, 128.11, 132.20, 132.73, 150.82, 167.31; MS (*m*/*z*, %): 279 (M**^+^**, 11.71%), 370 (100%); Anal. Calcd. for C_16_H_13_N_3_O_2_: C, 68.81; H, 4.69; N, 15.05. Found: C, 68.67; H, 4.55; N, 14.99%.

#### 3.1.4. General procedure for the Synthesis of Compounds **13a**–**c**

A mixture of **12** (2.79 g, 0.01 mol) and the corresponding isocyanate and/or isothiocyanate derivative (0.01 mol) was refluxed in dioxane (30 mL) for 5 h. The solid separated while hot was filtered, dried and crystallized from DMF/MeOH.

*2-{4-[4-(4-Chlorophenyl)-3-methyl-5-oxo-1,2,4-triazolidin-3-yl]phenyl}isoindoline-1,3-dione* (**13a**). Yield 47%; m.p. 254–256 ^o^C; IR (KBr, cm^−1^): 3299, 3224 (2NH), 1785 and 1714 (2C=O, isoindoline-1,3-dione), 1671 (C=O); ^1^H-NMR (DMSO-*d*_6_) δ 2.50 (s, 3H, CH_3_), 7.31 (d, 4H, *J* = 8.8 Hz, Ar-H), 7.34 (d, 4H, *J* = 8.8 Hz, Ar-H), 7.54–7.67 (m, 1H, Ar-H), 7.88–8.15 (m, 3H, Ar-H), 8.96 (s, 1H, NH, D_2_O exchangeable), 11.54 (s, 1H, NH, D_2_O exchangeable); ^13^C-NMR (DMSO-*d*_6_) δ 27.70, 87.03, 118.17, 120.31, 120.55, 123.70, 125.86, 127.75, 128.90, 129.12, 130.70, 132.20, 139.20, 156.39, 167.31; MS (*m*/*z*, %): 432.5 (M**^+^**, 0.7%), 104(100%); Anal. Calcd. for C_23_H_17_ClN_4_O_3_: C, 63.82; H, 3.96; N, 12.94. Found: C, 64.02; H, 3.75; N, 13.11%.

*2-[4-(4-Ethyl-3-methyl-5-thioxo-1,2,4-triazolidin-3-yl)phenyl]isoindoline-1,3-dione* (**13b**). Yield 39%; m.p. 247–249 °C; IR (KBr, cm^−1^): 3259, 3158 (2NH), 1782 and 1713 (2C=O, isoindoline-1,3-dione), 1216 (C=S); ^1^H-NMR (DMSO-*d*_6_) δ 1.13 (t, 3H, *J* = 6Hz, CH_2_C*H*_3_), 2.50 (s, 3H, CH_3_), 3.61 (q, 2H, *J* = 6 Hz, *CH*_2_CH_3_), 7.88–7.98 (m, 5H, Ar-H), 8.06–8.23 (m, 3H, Ar-H), 9.15 (s, 1H, NH, D_2_O exchangeable), 11.54 (s, 1H, NH, D_2_O exchangeable); ^13^C-NMR (DMSO-*d*_6_) δ 15.03, 37.13, 40.59, 90.03, 123.70, 125.59, 127.38, 128.63, 132.20, 133.06, 147.70, 167.31, 182.18; MS (*m*/*z*, %): 366 (M**^+^**, 0.5%), 104 (100%); Anal. Calcd. for C_19_H_18_N_4_O_2_S: C, 62.28; H, 4.95; N, 15.29. Found: C, 62.41; H, 4.78; N, 15.33%.

*2-[4-(3-Methyl-5-thioxo-4-phenyl-1,2,4-triazolidin-3-yl)phenyl]isoindoline-1,3-dione* (**13c**). Yield 42%; m.p. >300 °C; IR (KBr, cm^−1^): 3437, 3164 (2NH), 1785 and 1714 (2C=O, isoindoline-1,3-dione), 1217 (C=S); ^1^H-NMR (DMSO-*d*_6_) δ 2.50 (s, 3H, CH_3_), 7.15 (t, 1H, *J* = 7.2 Hz, Ar-H), 7.33 (t, 2H, *J* = 7.6 Hz, Ar-H), 7,54 (d, 2H, *J* = 7.2 Hz, Ar-H), 7.88–7.99 (m, 4H, Ar-H), 8.06–8.19 (m, 4H, Ar-H), 9.45 (s, 1H, NH, D_2_O exchangeable), 11.54 (s, 1H, NH, D_2_O exchangeable); ^13^C-NMR (DMSO-*d*_6_) δ 14.44, 70.80, 123.92, 125.60, 127.36, 127.50, 132.02, 133.02, 133.5, 135.23, 137.63, 139.81, 147.49, 167.39, 179.39; MS (*m*/*z*, %): 414 (M**^+^**, 1.25%), 104 (100%); Anal. Calcd. for C_23_H_18_N_4_O_2_S: C, 66.65; H, 4.38; N, 13.52. Found: C, 66.42; H, 4.17; N, 13.55%.

#### 3.1.5. Synthesis of 2-[4-(3-Methyl-5-thioxo-1,2,4-triazolidin-3-yl)-phenyl]isoindole-1,3-dione (**14**)

To a solution of **9** (2.65g, 0.01 mol) in dioxane (30 mL), thiosemicarbazide (0.91 g, 0.01 mol) was added. The reaction mixture was refluxed for 3 h. The solid separated on hot was filtered, dried and crystallized from DMF/acetone. Yield 33%; m.p. >300 °C; IR (KBr, cm^−1^): 3417–3271 (3NH), 1781 and 1707 (2C=O, isoindoline-1,3-dione), 1222 (C=S); ^1^H-NMR (DMSO-*d*_6_) δ 2.35 (s, 3H, CH_3_), 7.48 (d, 2H, *J* = 8.4 Hz, Ar-H), 7.90, 7.91 (dd, 2H, *J* = 3.6 Hz, 2.8 Hz, Ar-H), 7.94, 7.96 (dd, 2H, *J* = 8 Hz, 2.8 Hz, Ar-H), 8.04 (s, 1H, NH, D_2_O exchangeable), 8.09 (d, 2H, *J* = 8 Hz, Ar-H), 8.35 (s, 1H, NH, D_2_O exchangeable), 10.31 (s, 1H, NH, D_2_O exchangeable); ^13^C-NMR (DMSO-*d*_6_) δ 14.44, 66.80, 123.92, 127.36, 127.50, 132.02, 133.02, 135.23, 137.63, 167.39, 179.40; MS (*m*/*z*,%): 338 (M**^+^**, 0.22%), 153 (100%); Anal. Calcd. for C_17_H_14_N_4_O_2_S: C, 60.34; H, 4.17; N, 16.65. Found: C, 60.52; H, 4.17; N, 16.84%.

#### 3.1.6. Synthesis of (*ZE*)-*N*ʹ-{1-[4-(1,3-Dioxoindolin-2-yl)phenyl]ethylidene}benzohydrazide (**15**)

A mixture of **9** (2.65 g, 0.01 mol) and benzohydrazide (1.36 g, 0.01 mol) was refluxed in dioxane (30 mL) for 7 h. The solid separated on hot was filtered, dried and crystallized from propanol. Yield 48%; m.p. 247–249 °C; IR (KBr, cm^−^^1^): 3330 (NH), 1781 and 1704 (2C=O, isoindoline-1,3-dione), 1672 (C=O); ^1^H-NMR (DMSO-*d*_6_) δ 2.56, 2.64 (2s, 3H, *syn* & *anti* CH_3_), 7.59–7.68 (m, 5H, Ar-H), 7.98–8.10 (m, 8H, Ar-H), 10.85 (s, 1H, NH, D_2_O exchangeable); ^13^C-NMR (DMSO-*d*_6_) δ 22.70, 123.95, 124.50, 127.30, 127.51, 128.41, 128.79, 129.40, 132.03, 133.11 135.25, 138.09, 147.79, 163.20, 167.36; MS (*m*/*z*, %): 383 (M**^+^**, 4.05%), 105 (100%); Anal. Calcd. for C_23_H_17_N_3_O_3_: C, 70.05; H, 4.47; N, 10.96. Found: C, 69.98; H, 4.25; N, 10.97%.

#### 3.1.7. Synthesis of 2-[4-(2-Methyl-2*H*-imidazol-2-yl)phenyl]isoindole-1,3-dione (**16**)

To a solution of **9** (2.65 g, 0.01 mol) in dioxane (30 mL), ethylenediamine (0.6 g, 0.01 mol) was added. The reaction mixture was refluxed for 4 h. The solution was allowed to cool; the solid separated was filtered, dried and crystallized from ethanol 95%. Yield (26%); m.p. 215–217 °C; IR (KBr, cm^−1^): 1781 and 1704 (2C=O, isoindoline-1,3-dione); ^1^H-NMR (DMSO-*d*_6_) δ 2.62 (s, 3H, CH_3_), 6.56 (d, 1H, *J* = 8.4Hz, Ar-H), 7.49–7.54 (m, 8H, Ar-H), 7.66 (d, 1H, *J* = 8.4Hz, Ar-H); ^13^C-NMR (DMSO-*d*_6_) δ 27.32, 66.81, 124.03, 127.53, 128.58, 129.24, 131.98, 135.33, 136.28, 136.49, 167.13; MS (*m*/*z*, %): 303 (M**^+^**, 5.6%), 80 (100%); Anal. Calcd. for C_18_H_13_N_3_O_2_: C, 71.28; H, 4.32; N, 13.85. Found: C, 71.08; H, 4.27; N, 14.01%.

#### 3.1.8. General Procedure for the Synthesis of Compounds **17a**–**c**

A mixture of **9** (2.65 g, 0.01 mol) and the corresponding phenyl hydrazine hydrochloride derivative (0.01 mol) was heated at reflux temperature in absolute ethanol (30 mL) for 5–8 h (monitored by TLC). The solid separated on hot was filtered, dried and crystallized from dioxane.

*2-{4-[1-(Phenyl-hydrazono)-ethyl]-phenyl}-isoindole-1,3-dione* (**17a**). Yield 49%; m.p. 185–187 °C; IR (KBr, cm^−1^): 3355 (NH), 1783 and 1711 (2C=O, isoindoline-1,3-dione); ^1^H-NMR (DMSO-*d*_6_) δ 2.30, 2.63 (2s, 3H, *syn* & *anti* CH_3_), 7.26–7.45 (m, 3H, Ar-H), 7.47 (d, 1H, *J* = 8.4 Hz, Ar-H), 7.64 (d, 1H, *J* = 8.8 Hz, Ar-H), 7.93–7.97 (m, 4H, Ar-H), 7.99 (m, 3H, Ar-H), 8.11 (d, 1H, *J* = 8.8 Hz, Ar-H), 9.87 (s, 1H, NH, D_2_O exchangeable); ^13^C-NMR (DMSO-*d*_6_) δ 13.27, 113.34, 119.49, 124.03, 127.53, 128.58, 129.24, 131.98, 132.01, 135.33, 136.28, 139.35, 150.80, 167.49; MS (*m*/*z*, %): 355 (M**^+^**, 9.26%), 250 (100%); Anal. Calcd. for C_22_H_17_N_3_O_2_: C, 74.35; H, 4.82; N, 11.82. Found: C, 74.28; H, 4.69; N, 12.03%.

*2-(4-{1-[(4-Methanesulfonyl-phenyl)-hydrazono]-ethyl}-phenyl)-isoindole-1,3-dione* (**17b**). Yield 51%; m.p. 208–210 °C; IR (KBr, cm^−1^): 3330 (NH), 1783 and 1714 (2C=O, isoindolne-1,3-dione);^1^H-NMR (DMSO-*d*_6_) δ 2.36, 2.63 (2s, 3H, *syn* & *anti* CH_3_), 3.12 (s, 3H, SO_2_CH_3_), 7.42 (d, 1H, *J* = 8.8 Hz, Ar-H), 7.50 (d, 1H, *J* = 8.2 Hz, Ar-H), 7.64 (d, 2H, *J* = 8.8 Hz, Ar-H), 7.75 (d, 1H, *J* = 8.2 Hz, Ar-H), 7.96–8.01 (m, 5H, Ar-H), 8.11 (d, 2H, *J* = 8.4 Hz, Ar-H), 9.98 (s, 1H, NH, D_2_O exchangeable); ^13^C-NMR (DMSO-*d*_6_) δ 13.77, 44.73, 112.88, 123.92, 126.42, 127.52, 127.56, 129.15, 130.20, 132.01, 132.10, 135.22, 136.26, 150.26, 167.44; MS (*m*/*z*, %): 433 (M**^+^**, 58.21%), 163 (100%); Anal. Calcd. for C_23_H_19_N_3_O_4_S: C, 63.73; H, 4.42; N, 9.69. Found: C, 63.58; H, 4.66; N, 9.73%.

*4-(N*ʹ*-{1-[4-(1,3-Dioxo-1,3-dihydro-isoindol-2-yl)-phenyl]-ethylidene}-hydrazino)-benzenesulfonamide* (**17c**). Yield 52%; m.p. 265–267 °C; IR (KBr, cm^−1^): 3428–3258 (NH_2_ and NH), 1784 and 1711 (2C=O, isoindoline-1,3-dione); ^1^H-NMR (DMSO-*d*_6_) δ 2.34, 2.51 (2s, 3H, *syn* & *anti* CH_3_), 7.1 (s, 2H, NH_2_, D_2_O exchangeable), 7.36 (d, 2H, *J* = 8.8 Hz, Ar-H), 7.49 (d, 2H, *J* = 8.8 Hz, Ar-H), 7.68 (d, 2H, *J* = 8.8 Hz, Ar-H), 7.96–8.12 (m, 6H, Ar-H), 9.88 (s, 1H, NH, D_2_O exchangeable); ^13^C-NMR (DMSO-*d*_6_) δ 13.65, 112.56, 123.92, 126.32, 127.57, 127.71, 131.93, 132.03, 134.21, 135.22, 138.89, 142.89, 148.90, 167.47; MS (*m*/*z*, %): 434 (M**^+^**, 32.06%), 88 (100%); Anal. Calcd. for C_22_H_18_N_4_O_4_S: C, 60.82; H, 4.18; N, 12.90. Found: C, 60.69; H, 4.17; N, 12.73%.

### 3.2. Biological Testing

#### 3.2.1. Antimicrobial Sensitivity Test Using Agar Diffusion Method (Cup Technique)

The cup plate method is an accepted method when test samples diffuse from the cup through an agar layer in a Petri dish or plate to such an extent that the growth of added microorganisms is completely restricted to a circular area or zone around the cavity containing the solution of an antibiotic substance [23]. The anti-microbial activity was expressed as zone diameter in millimetres, which is measured by a ruler [24].

##### Microorganisms

Gram-positive organisms used in the study were *Staphylococcus aureus* LMG 3242, *Sarcina lutea*, *Enterococcus faecalis* OS4, *Listeria innocua* LMG 2710, *Mycobacterium pheli* and *Bacillus subtilis*. Gram-negative organisms used were *Escherichia coli* ATCC 5087, *Escherichia coli* ATCC 25922, *Proteus vulgaris* and *Pseudomonas aeruginosa*. The yeast used was *Candida albicans*.

##### Standard Antimicrobials

Gentamicin (CN), Cefotaxime (CTX), Ampicillin (AMP), Fluconazole (FLU) and Tioconazole (TIO) were all were prepared in concentration of 10 mg/mL.

##### Preparation of Sample Stock Solutions

All test samples (**10a**–**c**, **11a**–**c**, **12**, **13a**–**c**, **14**, **15**, **16**, **17a**–**c**) were dissolved in DMSO to give a final concentration of 10 mg/mL.

##### Experimental Procedure

Each overnight culture of the tested microorganisms was mixed with Muller Hinton agar media to give a final concentration of 1% microorganism (about 0.5 McFarland) and poured into sterile Petri dishes in a fixed amount of 20 mL under aseptic conditions. A sterile cork borer was used to prepare cups of 10 mm diameter. Test samples and standard drugs with volumes of 60 μL were introduced into the cups using a micropipette. All the plates were kept at room temperature for effective diffusion of the test drug and standard and incubated at 37 ± 1 °C for 24 h. The presence of inhibition zones around the cup indicated antibacterial activity. The diameter of the zone of inhibition was measured and recorded [25]. The percentage inhibition activities of tested compounds relative to standard antibiotics were calculated by applying this equation:

The percentage inhibition = (Zone of inhibition of tested compound − cup diameter)/(Zone of inhibition of standard antibiotic − cup diameter) × 100

##### Determination of Minimum Inhibitory Concentration (MIC) Using Agar Dilution Method According to Clinical Laboratory Standards Institute (CLSI)

For each sample, different concentrations were diluted with Muller Hinton agar to give a final concentration ranging from (200 µg/mL–0.7 µg/mL). DMSO was used as negative control plate. All bacterial isolates were subcultured on Brain Heart Infusion agar (B.H.I.A.) and incubated at 37 °C for 24 h [26,27]. Three colonies of each microorganism were suspended in 5 mL saline, and the suspension was adjusted to 0.5 McFarland standards and then diluted 10-fold with saline to give an organism suspension of (1 × 10^6^ to 5 × 10^6^ CFU/mL). This suspension was then further diluted by putting 1 mL suspension in 9 mL saline to give a final suspension volume of 1 × 10^5^ to 5 × 10^5^ CFU/mL. A multiple inoculator was used to inoculate the prepared agar plates. A 100 μL (*i.e.*, 10^4^ CFU) of the prepared inoculums were put in the well of a multi-inoculator, where each inoculation time by multi-inoculator gave about 10 μL of prepared inoculums per plate (*i.e.*, 10^3^ CFU). Each experiment was performed in duplicate. All plates were incubated at 35 °C for 48 h. The results were recorded in terms of MIC, which is the lowest concentration of antimicrobial/antifungal agent causing almost complete inhibition of growth or giving no visible growth.

#### 3.2.2. Anti-Oxidant Testing

##### Determination of Oxygen Radical Absorbance Capacity (ORAC)

Oxygen radical absorbance capacity (ORAC) was determined as described previously [28]. Briefly, 100 μL of 500 nM fluorescein and 25 μL of diluted solutions of tested compounds were pipetted into each working well of a microplate (96 well) preincubated at 37 °C. 25 μL of 250 mM AAPH was then added and the microplate was shaken for 5 s in a fluorometer Infinite 200 (Tecan, Mannedorf, Switzerland). The fluorescence (Ex. 485 nm, Em. 510 nm) was read every 2 min for 60 min. The net area under the curve was used to calculate ORAC which was expressed as a ratio between tested compound and trolox on an equimolar basis.

#### 3.2.3. Anti-Inflammatory Testing

##### Cell Culture

Stock solutions (10 mmol/L) of tested compounds were prepared by dissolving an appropriate quantity of each substance in DMSO. Dulbecco’s modified Eagle’s medium (DMEM, RPMI 1640 medium), foetal bovine serum (FBS), l-glutamine, penicillin and streptomycin were purchased from Sigma (St. Louis, MO, USA). Calcein AM was obtained from Molecular Probes (Life Technologies, Carlsbad, CA, USA).

The screening cell lines (T-lymphoblastic leukaemia cell line CEM, breast carcinoma cell line MCF7, cervical carcinoma cell line HeLa and human fibroblasts BJ) were obtained from the American Type Culture Collection (Manassas, VA, USA). CEM cell line were cultured in RPMI 1640 medium and the others in DMEM medium (Sigma); both media supplemented with 10% foetal bovine serum, 2 mmol/L l-glutamine, 10,000 U penicillin and 10 mg/mL streptomycin. The cell lines were maintained under standard cell culture conditions at 37 °C and 5% CO_2_ in a humid environment. Cells were subcultured twice or three times a week using the standard trypsinization procedure.

Human Umbilical Vein Endothelial Cells (HUVEC) were cultured in ECGM medium (Endothelial Cell Growth Medium, Provitro, Berlin, Germany), supplemented with 10% foetal bovine serum (Sigma-Aldrich, Munich, Germany). Cells were maintained under standard cell culture conditions at 37 °C and 5% CO_2_ in a humid environment. Cells were subcultured twice or three times a week using the standard trypsinization procedure. HUVECs were a kind gift from Prof Jitka Ulrichová (Medical Faculty, Palacky University, Olomouc, The Czech Republic).

##### CD62E (E-Selectin, ELAM)-induction Assays

Each well of the 96-well plates were coated with collagen G by applying 200 µL for 15 min at 37 °C. Outer wells (A1-A12, H1-H12, 1-H1 and A12-H12) contained only 200 µL/well medium and served as an evaporation barrier. 1 × 10^4^ HUVECs (Human Umbilical Vein Endothelial Cells) were seeded in each of the other wells in 200 µL medium and grown for 48 h to optimal confluence. Increasing concentrations of compounds were then added to the HUVEC-containing wells in triplicates, and the cells were incubated for 30 min, after which 10 ng/mL TNFα was added per well to stimulate NF-κB, and thus ELAM. After another 4 h incubation, the levels of ELAM in each of the HUVEC-containing wells were determined by enzyme-linked activity assays (ELISAs) as described below.

##### Cell-Surface ELISA ELAM

Cells were washed once with PBS and fixed with 0.1% glutaraldehyde (Sigma-Aldrich), for 15 min at room temperature. The cells were then washed 3 times with 200 µL per well PBS/0.05% Tween 20, blocked with 200 µL/well 5% BSA/PBS for 1 h, and washed again 3 times with 200 µL per well PBS/0.05% Tween 20. Anti-ELAM-antibody (clone BBA-1, R & D Systems, Minneapolis, MN, USA) diluted 1:5000 in 0.1% BSA/PBS (100µL per well) was then added for 1 h at room temperature and washed 5 times with 200 µL per well PBS/0.05% Tween 20. Subsequently, goat anti mouse-HRP antibody (Sigma-Aldrich) diluted 1:10000 in 0.1% BSA/PBS (100 µL per well) was applied and the cells were incubated for 1 h in the dark at room temperature and, after decanting, washed 5 times with 200 µL per well PBS/0.05% Tween 20. The HRP-activity of the cells in each of the wells was estimated using Fast-OPD (*o*-phenylenediamine dihydrochloride) (Sigma-Aldrich) assay as described [29] and absorbance was measured at OD_492nm_ in a plate reader (Tecan).

#### 3.2.4. Cytotoxicity Testing

For the ELAM expression assay the toxicity of tested compounds was assessed in HUVECs by Calcein AM (Molecular Probes, Invitrogen, Karlsruhe, Germany) cytotoxicity assays in 96-well microtiter plates [30]. 20 µL portions of each of the compound concentrations were added in triplicate to the cells, which were then incubated at 37 °C in an atmosphere containing 5% CO_2_ for 4 h, after which Calcein AM solution was added for 1 h according to the manufacturer’s instructions. The fluorescence of viable cells was quantified using a Fluoroskan Ascent instrument (Lab-systems, Vantaa, Finland) reader and on the basis of triplicate experiments the cytotoxic concentrations were calculated. Cytotoxicity of tested compounds was determined also in HUVEC after 24 h and in CEM, MCF7, HeLa and BJ after 72 h by Calcein AM assays as described above.

##### Molecular Modeling and Docking

The celecoxib-COX-2 crystal structure was obtained from (PDB: ID 3LN1) [21]. Docking of the ligand was carried out. The root mean square deviation was 0.50 Å. Docking was performed using London dGforce. Force field energy was used to refine the results. The most active compound **17c** was docked by MOE after preparation of the selected compound through its 3D protonation and selecting the least energetic conformer. The same docking method used for both ligand and **17c**. Amino acid interactions and hydrogen bond lengths were measured and summarized in (Table 4).

## 4. Conclusions

The tested compounds in this study were evaluated against Gram positive, Gram negative and Fungi. The most active phthalimide derivative as an anti-microbial agent was hydrazonoethyl-phenylisoindoline-1,3-dione **12**. Most phthalimide derivatives showed antimicrobial activity with the exception of **10a** and **10b** and **13a** and **10** **b** on Gram negative bacteria, in addition to **13a** which had no action on *Candida albicans.* None of the tested phthalimide derivatives had any cytotoxic activity. Two, **13b** and **13c** (ethyl and phenyl isothiocyanate derivatives) had anti-oxidant activity. Phthalimide derivatives had mild anti-inflammatory activity *in vitro* albeit a strong inhibition of E-selectin by **17c**.
